# Editorial: Targeting triple negative breast cancer by natural compounds

**DOI:** 10.3389/fphar.2023.1172245

**Published:** 2023-03-17

**Authors:** Shashank Kumar, Sanjay Gupta, Subash Chandra Gupta

**Affiliations:** ^1^ Department of Biochemistry, School of Basic Science, Central University of Punjab, Bathinda, India; ^2^ Department of Urology, Case Western Reserve University, Cleveland, OH, United States; ^3^ Department of Biochemistry, Institute of Science, Banaras Hindu University, Varanasi, India; ^4^ Department of Biochemistry, All India Institute of Medical Sciences, Guwahati, Assam, India

**Keywords:** triple-negative breast cancer (TNBC), phytochemical, natural product, anti-cancer, therapeutics

Breast cancer is the most prevalent cancer in women globally and a major global health concern. One in every twelve women worldwide will be diagnosed with this malignancy at some point in their lifetime. Breast cancer is a heterogeneous disease with diverse clinical and molecular characteristics. Treatment decisions for advance-stage breast cancer are guided by the expression of three major therapeutic targets, *viz.* estrogen receptor-α (ER), progesterone receptor (PR), and human epidermal growth factor receptor-2 (HER-2) (Shuaib et al., 2022). While ER-positive breast cancers are treated with ER antagonists such as tamoxifen and aromatase inhibitors, HER2-positive tumors are treated with HER2 inhibitors such as herceptin. Triple-negative breast cancers (TNBC) that do not express these receptors account for 15%–24% of all breast cancers. The prevalence of TNBC seems to be on the rise in developing nations (Thakur et al., 2018). TNBC treatment is difficult because of disease’s aggressiveness, poor clinical prognosis, and propensity for relapse. It has been extremely difficult to develop more effective treatments for TNBC because of the lack of actionable targets (Diana et al., 2020). Although chemotherapy with taxanes is still a standard-of-care treatment for advanced-stage TNBC, the response is typically short, linked to chemo-resistance, and has a dismal prognosis, with a median overall survival of 9–12 months. As such, strategies for early prevention and/or treatment are needed. Natural agents are known for their role in disease prevention and therapeutic potential, which substantiates the importance of these molecules in human life (Prajapati et al., 2022). Natural products are well known for their minimal side-effect and cost effectiveness. Natural compound-based standalone or combination therapy could be utilized for the better management of triple-negative breast cancer (Kushwaha et al., 2019). The present Research Topic, “Targeting Triple Negative Breast Cancer by Natural Compounds,” has assembled four articles, including original research articles contributed by researchers working in the area of triple-negative breast cancer and its treatment by natural compounds.

In the present research article Wei et al. studied the effect of Cordycepin (an active phyto-constituent present in Cordyceps mushroom) on metastasis modulation in mouse TNBC model. The study highlighted that Cordycepin inhibits cellular growth and decreases migration and invasion potential in TNBC cells (BT549 and 4T1 cells) at micromolar concentrations. Mechanistically the study showed that Cordycepin treatment of TNBC cells reversed the expression profile of epithelial to mesenchymal transition markers E-cadherin (increased) and N-cadherin (decreased) in concentration dependent manner. Further, the study showed that Cordycepin treatment reduces the expression of EMT-related transcription factors (SNAIL, SLUG, TWIST1, ZEB1, and ZEB2) in TNBC cells significantly compared to non-treated cells. Furthermore, the authors utilize the 4T1 mouse allogenic tumor model to study the metastasis inhibition potential of Cordycepin *in vivo*. The immune system plays an important role in cancer metastasis and thus selection of the animal experimental model with the intact immune system provides decent translational pre-clinical data on the anti-metastatic potential of Cordycepin. Overall, the study highlights Cordycepin as a natural lead compound managing metastasis and invasion in mouse TNBC model.

In the next research article Wang et al. studied the effect of Ailanthone (phytochemical isolated from *Ailanthus altissima*) on bone metastasis in TNBC and attempted to search for the underlying mechanism(s). Interaction between breast cancer cells (by secreting osteolytic factors) and bone microenvironment is largely involved in breast cancer metastasis. Interestingly, the study focused on the effect of Ailanthone on osteoclast differentiation which was induced by TNBC cells mediated cytokine secretion. The study reported that Ailanthone treatment can potentially inhibit osteoclasts production by inhibiting the osteolytic factors secretion in RANKL-dependent manner, thereby inhibiting the downstream molecular signaling pathways (PI3K/AKT, NF-κB, and MAPK) involved in TNBC cell metastasis. Overall, these findings reveal a natural compound mediated novel therapeutic strategy for managing breast cancer metastases.

In the following article, Zhou et al. studied the effect of Polyphyllin III or dioscin (*Paris polyphylla* rhizome saponin) in *vitro* TNBC model. The study also explored the underlying anticancer mechanism(s) of this natural compound. Polyphyllin III has the potential to induce ferroptosis in TNBC cells at micromolar concentration. Studies were performed using the transmission electron microscopy-based morphological assessment of the mitochondrial membrane/cristae density ratio in TNBC cells. Further examination of hallmarks of ferroptosis, including accumulation of cellular lipid reactive oxygen species and decrease in GSH levels in Polyphyllin III, treated TNBC cells, substantiates these findings. The study proposed that increasing the expression of Acyl-CoA synthetase, a long chain family member 4 (ACSL4), by Polyphyllin III treatment might be a possible ferroptosis inducing mechanism in TNBC cells. In an important observation, the study pointed out that KLF4-mediated overexpression of xCT in TNBC cells acts as a protective response during ferroptosis in TNBC cells in response to therapeutic drugs and may result in enhancing drug resistance. The study proposed that this protective response could be diminished by using xCT inhibitors combined with Polyphyllin III to counter balance the protective response in TNBC cells during ferroptosis. The study also showed that the combination treatment significantly increased Polyphyllin III treatment susceptibility in TNBC cells.

The last research article by Zhang et al. studied the effect of Ursolic acid, a naturally occurring triterpenoid acid, in *in vitro* and *in vivo* TNBC models. The author strikingly utilized a combination of computer-based and pre-clinical experiments to decipher the anti-TNBC effect of Ursolic acid and its underlying mechanism(s). The compound has the potential to reverse the drug-resistance in cancer cells. The study further proved that Ursolic acid can significantly inhibit the proliferation of TNBC cells. The compound selectively kills TNBC cells, compared to normal cells. Furthermore, Ursolic acid has the potential to arrest the cells in the G2/M phase, reduce migration/invasion (by decreasing MMP2 and MMP9 levels), and induces apoptosis (by altering the expression of Bcl2, cleaved caspase-3 and -9). Pharmacology-based network analysis identified the enrichment of PLK1 and CCNB 1 in the p53 signaling pathway in TNBC cells. Treatment of TNBC-xenograft mice with Ursolic acid resulted in a protective effect and reduced tumor growth through the PLK1-CCNB1-p53 axis.

In this Research Topic, some natural compounds are highlighted with the mechanisms of action, demonstrating their effectiveness against triple-negative breast cancer. Future research focusing on more detailed studies can open new horizons in breast cancer prevention and/or treatment, which could enhance the overall survival and quality-of-life of breast cancer patients.
